# Supplementary material for: Long-Term Impact of Early-Life Stress on Hippocampal Plasticity: Spotlight on Astrocytes
Source: Int J Mol Sci. 2020 Jul 15;21(14):4999. doi: 10.3390/ijms21144999 (PMC7404101; doi:10.3390/ijms21144999)
Supplement: Supplementary file 1 [file ijms-21-04999-s001.pdf]

Supplementary Information

Methods

Animals. Wistar rats (~3 weeks-old) were obtained from Harlan, (Germany) and were habituated to our animal facility for 1 week before performing behavioral experiments. Juvenile stress (JS) was commenced using a 3-day variable stress protocol (PND 27-29) as described previously [1–3]. Thereafter, stressed animals were left undisturbed in their home cage. Control animals remained in their home cage for the whole period until electrophysiology experiments were performed. All experiments were conducted in accordance with the European and German regulations for animal experiments and were approved by the local authorities (LAGESO, T0068 / 02 and G0397 / 09).

Electrophysiology. Adult male Wistar rats (PND 74), with or without a history of JS, were deeply anesthetized with a combination of isoflurane and laughing gas (N_2O) and decapitated. Brain was rapidly removed and placed in cold (4-8 C) carbogenated (5% CO2/ 95% O2) artificial cerebrospinal fluid (aCSF) containing (in mM) 129 NaCl, 21 NaHCO3, 3 KCl, 1.6 CaCl2, 1.8 MgSO4, 1.25 NaH2PO4 and 10 glucose. Ventral transverse-like brain slices (400 µm) were obtained from the temporal pole by cutting horizontal slices at an angle of about 12°. Three to four most ventral slices were transferred to an interface chamber perfused with aCSF at 36 ± 0.2°C (flow rate: 1.8 ± 0.2 ml / min, pH 7.4, osmolarity ~300 mosmol/kg). Slices were incubated at least for 90 min before starting recordings. Extracellular field recordings were obtained by placing a glass electrode filled with ACSF (1-4 MΩ) in the middle of the dendritic molecular cell layer at 70-100 µM depth. Bipolar stainless steel stimulation electrode (electrode impedance in ACSF: ~10 KOhm; tip separation: 100-200 µm) was placed in the middle one-third of the molecular layer to stimulate medial perforant pathway (PP) [4]. Correct positioning of electrodes was confirmed by depression of field excitatory postsynaptic potentials (fEPSPs) upon a paired-pulse stimulation at 40-50 ms interpulse interval [5]. Baseline transmission was assessed via obtaining an input-output (I-O) curve using stimulation intensities from 1 to 5 V. The stimulus intensity that resulted in 50 % of the maximum fEPSP amplitude was further used for the paired-pulse (PP) and long-term potentiation (LTP) experiments. PP responses were recorded using interpulse intervals from 10 ms to 1000 ms. After 10 min of baseline recording (pulse duration: 100 µs; interval: 0.033Hz), LTP was induced using a single train of 100 pulses for 1 s ((pulse duration: 200 µs; 100 Hz). After LTP induction responses were recorded for 40 min (0.033 Hz). Signals were pre-amplified using a custom-made amplifier and low-pass filtered at 3 kHz. Signals were sampled at a frequency of 10 kHz and stored on a computer hard disc for off-line analysis.

Data Analysis. Data were analyzed offline using Spike2 (version 8, CED). For the analysis of fEPSPs, ascending peak-to-peak amplitude (mV) was calculated. The paired-pulse ratio was

measured by dividing the peak-to-peak amplitude of the second fEPSP by the first fEPSP. For the analysis of the LTP data, the data was normalized to the average of 10 min baseline before the induction of LTP.

Statistical Analysis. Data were reported as mean ± standard error of the mean (SEM). Before statistical comparison of different groups (control vs. JS) normality test (Shapiro-Wilk Test) and equal variance test was performed. I-O curves and paired-pulse responses were statistically compared using two-way repeated ANOVA. The group difference for LTP was assessed by comparing the average of normalized values at 30-40 min after LTP induction using two-tailed Student's T-test (SigmaPlot for Windows Version 11.0, 2008, Systat software).

Results

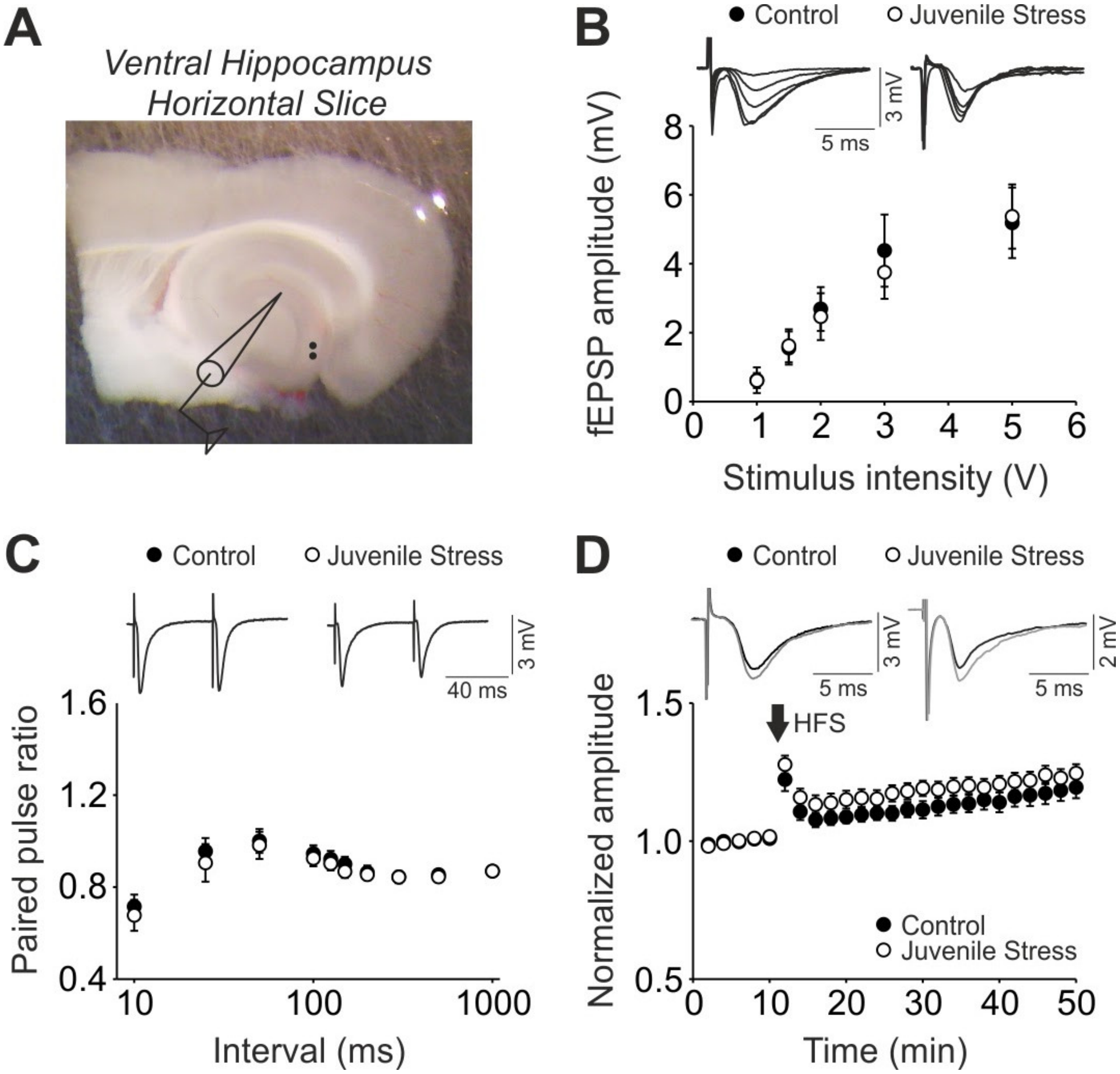

Supplementary Figure. Juvenile stress does not change synaptic transmission and plasticity at medial PP-to-DG synapse at adult age (N=5 control rats and N=4 JS rats). **(A)** A horizontal brain slice including a transverse-like section of the hippocampus. The position of the stimulation electrode (2 black dots) and the recording electrode are depicted. No significant differences were observed in **(B)** baseline synaptic transmission (F(1, 18) = 0.0221, p=0.883; Control: n=10 slices and Juvenile Stress: n=10 slices), **(C)** STP (F(1, 23) = 0.566, p=0.459; Control: n=12 slices and Juvenile Stress: n=13 slices) and **(D)** HFS-induced LTP (T(18) = -1.100, p=0.286; Control: n=9 slices and Juvenile Stress: n= 11 slices) between animals with a history of juvenile stress and control animals.

References

1. Albrecht, A.; Ivens, S.; Papageorgiou, I.E.; Çalışkan, G.; Saiepour, N.; Brück, W.; Richter-Levin, G.; Heinemann, U.; Stork, O. Shifts in excitatory/inhibitory balance by juvenile stress: A role for neuron-astrocyte interaction in the dentate gyrus. *Glia* **2016**, *64*, n/a-n/a, doi:10.1002/glia.22970.

2. Ivens, S.; Çalışkan, G.; Papageorgiou, I.; Cesetti, T.; Malich, A.; Kann, O.; Heinemann, U.; Stork, O.; Albrecht, A. Persistent increase in ventral hippocampal long-term potentiation by juvenile stress: A role for astrocytic glutamine synthetase. *Glia* **2019**, *67*, 2279–2293, doi:10.1002/glia.23683.

3. Gruber, D.; Gilling, K.E.; Albrecht, A.; Bartsch, J.C.; Çalişkan, G.; Richter-Levin, G.; Stork, O.; Heinemann, U.; Behr, J. 5-HT receptor-mediated modulation of granule cell inhibition after juvenile stress recovers after a second exposure to adult stress. *Neuroscience* **2015**, *293*, doi:10.1016/j.neuroscience.2015.02.050.

4. Petersen, R.P.; Moradpour, F.; Eadie, B.D.; Shin, J.D.; Kannangara, T.S.; Delaney, K.R.; Christie, B.R. Electrophysiological identification of medial and lateral perforant path inputs to the dentate gyrus. *Neuroscience* **2013**, *252*, 154–168, doi:10.1016/j.neuroscience.2013.07.063.

5. Annamneedi, A.; Caliskan, G.; Müller, S.; Montag, D.; Budinger, E.; Angenstein, F.; Fejtova, A.; Tischmeyer, W.; Gundelfinger, E.D.; Stork, O. Ablation of the presynaptic organizer Bassoon in excitatory neurons retards dentate gyrus maturation and enhances learning performance. *Brain Struct. Funct.* **2018**, *223*, 3423–3445, doi:10.1007/s00429-018-1692-3.
